# Comparison of the effects of hyaluronidase and hyaluronic acid on probiotics growth

**DOI:** 10.1186/1471-2180-13-243

**Published:** 2013-11-04

**Authors:** Alessandro Di Cerbo, Maria Aponte, Rita Esposito, Moreno Bondi, Beniamino Palmieri

**Affiliations:** 1 Dipartimento di Chirurgia Generale e Specialità Chirurgiche, Università degli Studi di Modena e Reggio Emilia, via del pozzo 71 41124, Modena, Italy; 2 Istituto di Scienze dell’Alimentazione, ISA-CNR, Via Roma, 64, 83100, Avellino, Italy; 3 Dipartimento di Scienze della vita, Università degli Studi di Modena e Reggio Emilia, Via Campi 287, 41125, Modena, Italy

**Keywords:** Hyaluronic acid, Hyaluronidase, Lactic acid bacteria

## Abstract

**Background:**

Hyaluronic acid has several clinical applications. Recent evidences suggested antimicrobial properties against several pathogens. The aim of the present survey was to evaluate the effect of hyaluronic acid, alone or in combination with hyaluronidase, on protechnological or probiotic strains.

**Results:**

The role of hyaluronic acid and hyaluronidase on *in vitro* growth rate of different lactic acid bacteria was investigated. Standard methods revealed that low concentrations of hyaluronic acid (0.5-0.125 mg ml^-1^), and hyaluronidase at fixed concentration (1.6 mg ml^-1^), resulted in an increased bacterial strains growth up to 72 hours whereas higher concentrations of the acid (2 and 1 mg ml^-1^), and hyaluronidase at the same fixed concentration, reduced the bacterial growth.

**Conclusions:**

Observations might suggest a possible protective role of both hyaluronidase and low doses of hyaluronic acid towards some strains, supporting their *in vivo* proliferation and engraftment after oral administration. Hyaluronidase introduction into growth medium greatly enhanced the bacterial growth up to 72 hours.

## Background

Hyaluronic acid (HA), a large linear glycosaminoglycan which is mostly present within extracellular matrix and whose molecular weight ranges from 8 × 10^5^ (LMWHA) to 2 × 10^6^ (HMWHA) Da [1], is a chain of repeating disaccharide units of D-glucuronic acid and N-acetyl-D-glucosamine [2]. HA is involved in biological and pathological processes such as cell adhesion, migration, proliferation, differentiation [3], vascular diseases and lymphocyte trafficking [4,5].

HA Anti-inflammatory action [6,7], bacteriostatic effect [8] and antioxidant properties [9] have been recently highlighted with a wide range of potential therapeutic perspectives such as oral, pneumological, dermatological and urological areas [10]. Healing properties of degradation products of HA achieved by N-acetylglucosaminic bonds breakdown, catalysed by the hyaluronidases, have been also well described in the literature [11]. Hyaluronidase (Hy), “hydrolases” with a molecular weight of approximately 60000 Da, has been widely used in medicine due to its ability to reduce extravasation injuries [12], to temporarily liquefy hyaluronic acid increasing the permeability of vessel membranes [13] and, as recently observed in Watanabe heritable hyperlipidaemic rabbits, to cause a partial disruption of the atherosclerotic plaque surfaces [14]. The hyaluronidases can be subdivided into three types [15]: 1) hyaluronate-4-glycanohydrolases (EC 3.2.1.35), that are present in mammalian spermatozoa, lysosomes and the venoms of various insects and snakes; 2) hyaluronate-3-glycanohydrolases (EC 3.2.1.36), that are produced by leeches and some hookworms and 3) bacterial hyaluronidases or hyaluronate lyases (EC 4.2.2.1 or EC 4.2.99.1).

Commonly used hyaluronidases are the partially purified bovine and ovine testicular ones. In spite of such a wide employment of both HA and Hy, only a few studies have been conducted to assess their possible combined effects, if any, on protechnological or probiotic bacteria. Based on the survey of Ardizzoni et al. (2011) [8], focused on the inhibitory effect of HA on a group of pathogenic bacteria and fungal strains, the aim of the present study was to evaluate the effects of HA on potential probiotic Lactic Acid Bacteria (LAB).

## Results and discussion

LAB engraftment within human gut has been the main challenge of last decade. However, well standardized procedures to achieve a long lasting engraftment still lack. This study, has been focused upon HA- Hy - LAB interaction to promote bacterial engraftment and feeding in order to enhance and prolong their beneficial effects. Firstly, the antimicrobial effect of HA was evaluated by MIC test in MRS agar. Among strains listed in Table 1, no one proved to be inhibited by HA even at a concentration of 4 mg ml^-1^. pH values of HA dilutions ranged from 6.5 to 7.6, corresponding to an HA concentration of 4 and 0.0625 mg ml^-1^, respectively. Moreover, when *Lactobacillus* (*Lb.*) *rhamnosus* LbGG cells were exposed, for 30 min, to different levels of HA (4–0.0625 mg ml^-1^) a slight increase (about 0.5 log CFU ml^-1^) in microbial counts was recorded (data not shown). In other words, high molecular weight HA did not exert any antimicrobial activity when tested on several LAB strains, but, on contrary, it seemed to enhance the bacterial viability.

**Table 1:** Strains used in this study and source of isolation

**Taxon**	**Strain**	**Source**	**Reference**
*Lb. rhamnosus*	LbGG	American Type Culture Collection	ATCC53103
*Lb. casei*	491	Provolone del Monaco cheese	[16]
*Lb. casei*	496	Provolone del Monaco cheese	[16]
*Lb. pentosus*	OM13	Table olives	[17]
*Lb. rhamnosus*	VT1	Parmigiano Reggiano cheese	[18]
*Lb. rhamnosus*	RBM526	Parmigiano Reggiano cheese	[18]
*Lb. rhamnosus*	RBT739	Parmigiano Reggiano cheese	[18]
*St. macedonicus*	67	Provolone del Monaco cheese	[19]
*St. thermophilus*	309	Provolone del Monaco cheese	[19]
*St. thermophilus*	247	Provolone del Monaco cheese	[19]
*St. thermophilus*	82A	Provolone del Monaco cheese	[19]

To better understand the - strains viability improvement, the ability to ferment HA and its precursor was evaluated by assessment of pH lowering according to a conventional procedure. All tested strains, namely three urease positive streptococci [19] and LbGG, proved to be able to utilize N-acetyl-D glucosamine, but not D-glucuronic acid as well as HA.

LbGG is a probiotic strain able to survive to 30 min of exposure to simulated gastric juice but not to 90 min [20]. Strain’s survival, evaluated in presence of increasing concentration of HA (0.0125-1.6 mg ml^-1^) to simulated gastric juice for 90 min, highlighted a weak positive gastro-protective effect that appeared directly correlated to HA concentration: 1) At 1.6 and 0.8 mg ml^-1^ HA a five Log of reduction (from 7 to 2 CFU ml^-1^) was recorded; 2) At 0.4 and 0.2 mg ml^-1^ HA a 5.5 Log reduction (from 7 to 1.5 CFU ml^-1^) was recorded; 3) At HA concentration lower than 0.1 mg ml^-1^ no strain survival was detected. At the used concentrations, HA is not able to protect the probiotic strain *Lb. rhamnosus* GG during a 90 minutes long exposition to simulated gastric juice, but further studies would be useful to understand if results may be improved by considering higher concentration of HA.

**Figure 1:**
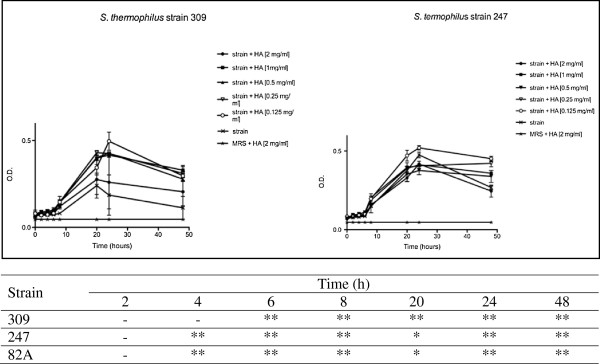
**Effects of HA on *St. thermophilus *strains 309 and 247 until 48 h.** Bacteria were employed at a starting concentration of 1 × 10^6^ CFU mL^-1^. Lower panel: statistical significance between HA-treated and untreated strains. **Highly significant (P < 0.01); *significant (P < 0.05); - not significant (P > 0.05).

A widely accepted *in vitro* system, which allows simultaneous evaluation of several HA doses, was compared with an innovative method based on the old concept of dynamic light scattering. By these two approaches comparable kinetic curves were obtained. Firstly, tests were performed on three selected urease positive strains belonging to *Streptococcus* (*St*.) *thermophilus* species in presence of growing concentrations of HA, until 48 h. As shown in Figure 1, each strain displayed a recurrent trend in the O.D. kinetics. In detail, curve profiles dropped after 24 h in all cases, showing a higher marked decrease when HA concentration was higher. When lower concentrations of HA were used, O.D. decrease was limited. Strain 82A behaved as 247 and therefore was not shown.

**Figure 2:**
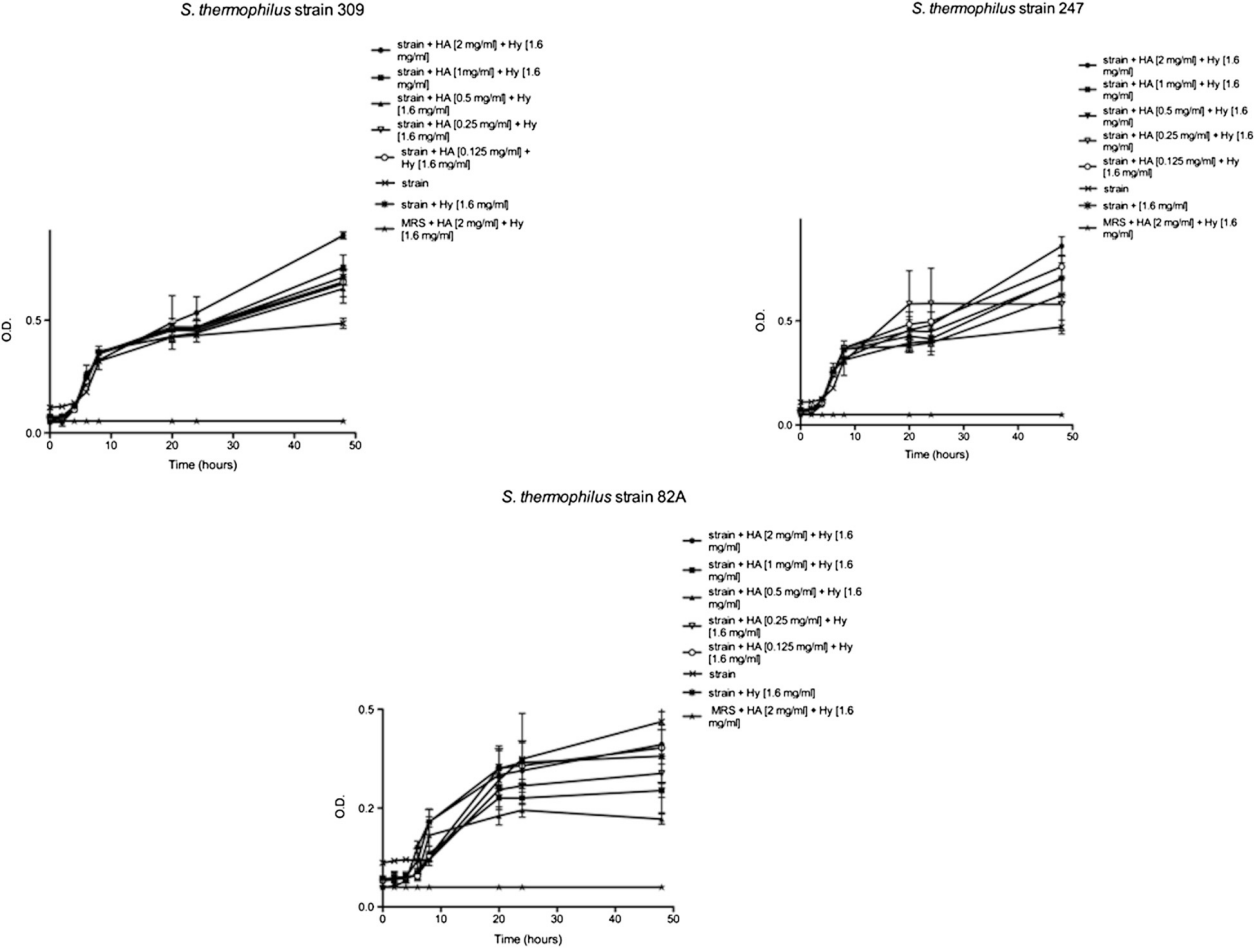
**Effects of HA and Hy on *St. thermophilus *309, 247 and 82A until 48 h.** Bacteria were employed at a starting concentration of 1 × 10^6^ CFU mL^-1^. Lower panel: statistical significance between HA-Hy-treated and untreated strains. **Highly significant (P < 0.01); *significant (P < 0.05); - not significant (P > 0.05).

Streptococci were even employed for the same set of trials previously described, but in presence of both HA and Hy. According to obtained data (Figure 2), strains displayed after 24 h a completely different behavior: strains 309 and 247 exhibited an O.D. increase, above all in presence of higher concentrations of HA, indicating a bacterial growth enhancement.

**Figure 3:**
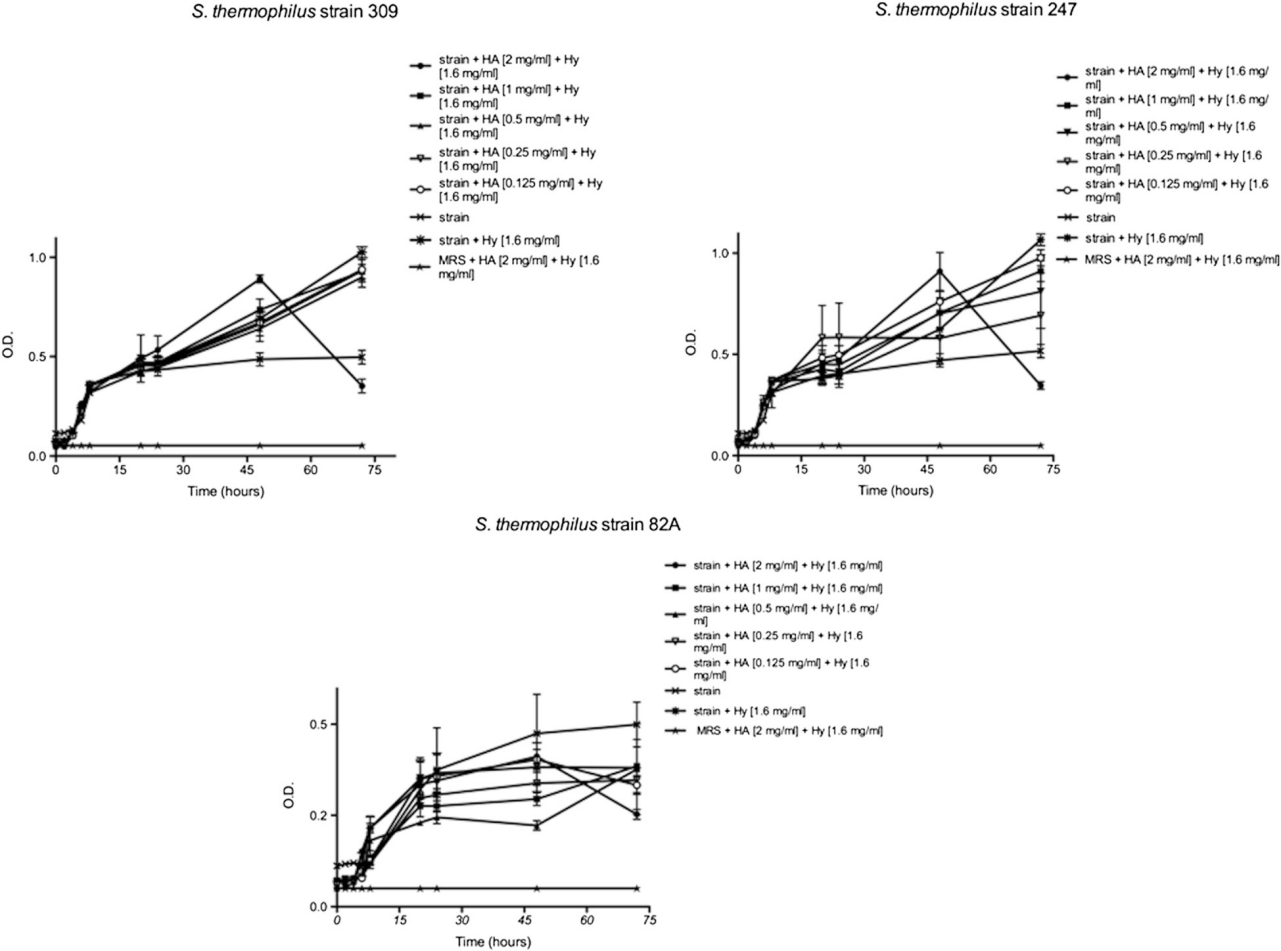
**Effects of HA and hy on *St. thermophilus *309, 247 and 82A until 72 h.** Bacteria were employed at a starting concentration of 1 × 10^6^ CFU mL^-1^. Lower panel: statistical significance between HA-Hy-treated and untreated strains. **Highly significant (P < 0.01); *significant (P < 0.05); - not significant (P > 0.05).

Monitoring strains’ activity up to 72 hours revealed a slight increase of the slope, except in presence of the highest HA concentration. As shown in Figure 3, each strain displayed the same trend at the highest HA concentration. The curve profile of each strain at 2 mg mL^-1^ of HA showed a slight decrease after 24 h as for higher HA concentration. At lower HA concentrations both a little O.D. increase for 82A strain and a slight O.D. increase for 309 and 247 strains were observed.

These preliminary experiments, demonstrated that bacterial growth may be influenced by HA concentration, by Hy concentration and by both of them.

Standard method indicated that a bacterial growth inhibition was observable when HA, along with Hy, was used at concentrations ranging from 2 to 1 mg ml^-1^. When considering higher HA concentrations (ranging from 0.5 to 0.125 mg ml^-1^), along with Hy, a growth stimulation up to 72 hours was observed. These results provide interesting insights about LAB growth kinetics, and highlight a possible synergistic role of the two challenged molecules that is likely to be related to the ability of LAB strains to use the N-acetyl-D glucosamine monomer as carbon source.

Although speculative, a possible combined role of HA and hyaluronidase on the bacterial growth was already hypothesized by Starr et al. (2006) [21]. Hy^-^*Streptococcus* (*St.*) *pyogenes* was shown to grow with N-acetylglucosamine but not with D-glucuronic acid as a sole carbon source. The same metabolic behavior was recorded in protechnological and probiotic LAB during this study. Only Hy^+^ strains could grow utilizing HA, as a sole carbon source, suggesting that Hy could permit the strain to utilize host HA as an energy source.

In conclusion, especially high HA concentrations seem to inhibit bacterial growth, however when low HA concentrations are combined with Hy the bacterial growth seems to be enhanced even beyond 72 hours.

Further studies, in order to understand if the effects of HA and Hy are strain specific as they seems to be, are urgently required; specifically, a wider screening of different LAB with interesting features, such as urease positive and/or hyaluronidase activity, might help to outline a new probiotic oral formula with enhanced prebiotic gut adherence properties and more effective therapeutic effect.

## Conclusions

The effect of hyaluronic acid on protechnological or probiotic bacteria has never been evaluated before. In this study, the effect of hyaluronic acid, alone or in combination with hyaluronidase, on three streptococci and one probiotic Lactobacillus strain was assessed. By obtained evidences, a synergistic role of the two molecules was described: when low hyaluronic acid concentrations are combined with hyaluronidase, the bacterial growth appeared greatly enhanced even beyond 72 hours. This phenomenon could be related to ability of tested strains to metabolize N-acetyl-D glucosamine, one of the precursor of hyaluronic acid.

## Methods

### Media and reagents

MRS (Oxoid LTD, Basingstoke, Hampshire, U.K.) was employed for bacterial strains growth, strain maintenance and viable count assessment. Sterile saline solutions of High Molecular Weight HA (1837 kDa, 8 mg ml^-1^) where kindly provided by IBSA (Institute Biochemique SA, Lugano, CH). Hyaluronidase solution (Jaluronidasi 100 I.U., 3.2 mg ml^-1^) was purchased from Farmacia Testi snc, Milan, Italy.

### Evaluation of minimal inhibitory concentration for HA

Dilutions for HA MIC determination were performed in sterile deionized water with concentrations ranging from 0.0625 up to 4 mg ml^-1^ for a total of 7 levels of exposure. 50 μl of each dilution were loaded into wells in MRS agar plates seeded with tested strains. pH values of HA solutions were evaluated by means of pH-meter (Beckman PHI43). LAB tested are reported in Table 1.

Tolerance to HA of strain *Lb*. *rhamnosus* LbGG (ATCC) was also evaluated. Briefly, strain was subcultured twice in MRS (incubation at 30°C). Cells in early stationary phase (7.91 ± 0.29 Log CFU ml^-1^) were collected by centrifugation (6.500 rpm, 10 min), washed once with sterile Ringer solution (Oxoid) and resuspended in the same saline. 200 μl of sterile water solutions of HA (0.0625, 0.125, 0.25, 0.5, 1, 2, 4 and 8 mg ml^-1^) were added to 200 μl of cell suspensions. Positive control was realized by adding 200 μl of sterile saline instead of HA. After 30 min of incubation at 37°C, living cells were enumerated by drop counting method (Collins et al., 1989) on MRS agar plates, followed by incubation for 72 h at 37°C.

### Effect of HA on Lb.GG tolerance to simulated gastric juice

The effect of HA on LbGG tolerance to simulated gastric juice was determined according to the procedure reported by Michida et al. (2006) [22]. Briefly, cells were harvested from cultures in exponential phase of growth by centrifugation (6.500 rpm, 10 min), washed twice with sterile saline (0.5%, w/v), and resuspended in the same sterile saline. Simulated gastric juice was prepared daily by suspending pepsin (1:10 000, ICN) in sterile saline (0.5%, w/v) to a final concentration of 3 g l^-1^ and adjusting the pH to 2.00 with concentrated HCl using a pH meter. Aliquots (0.2 ml) of the cell suspensions were transferred to a 2.0 ml capacity Eppendorf tube, mixed with 0.3 ml of sterile water solutions of HA (0.125, 0.25, 0.5, 1, 2, 4, and 8 mg ml^-1^) and finally mixed with 1.0 ml of simulated gastric. After incubation at 37°C for 90 min, cells viability was assayed by drop counting method [23] on MRS agar plates (incubation for 72 h at 30°C).

### LAB’s capability to utilize HA and its precursors as carbon sources

Acid formation by D-glucuronic acid, N-acetyl-D glucosamine, and HA was evaluated in MRS broth without glucose and meat extract with 2% added carbohydrates, as filter sterilized water solutions, and 0 · 004% chlorophenol red. Media were inoculated with cell suspensions in sterile saline solution (about 6 log CFU ml^-1^). Tests were performed on three urease positive *St. thermphilus* strains, namely 309, 82A and 247, and LbGG.

### Assessment of HA and Hy effect on LAB strains

The effect of HA and HA in combination with Hy was evaluated on three *St. thermophilus* urease positive strains (309, 247, and 82A). The assay was performed in 96-well microplates (Corning Inc., NY, USA). Firstly, 200 μl of HA + MRS [4, 2, 1, 0,5 and 0.25 mg ml^-1^] were added in triplicate in each plate. Then 10 μl of LAB cell suspensions (working concentrations of about 1 × 10^6^ CFU ml^-1^) in sterile saline solution were added. Uninoculated MRS was used as control. Plates were incubated at 37°C in an incubator (Ekort 1500, Angelantoni industrie, Milano, Italy). The O.D. values were measured at a wavelength of 595 nm at 0, 2, 4, 6, 8, 20, 24 and 48 hours by means of a microplate reader (Tecan, Austria).

For the evaluation of HA-Hy effect, the procedure above described was repeated by adding to each well 100 μl of Hy [1,8 mg ml^-1^ in a saline solution] and 10 μl of each strain (about 1 × 10^6^ CFU ml^-1^). O.D. values were measured at 0, 2, 4, 6, 8, 20, 24, 48 and 72 h of incubation at 37°C.

### Data analysis

Data obtained from the O.D. readings were used to draw charts where O.D. was expressed as a function of time. Each point of the curves is the average value of three replicates (subtracted of the blank) performed in the same experimental conditions. Statistical analyses were performed at 2 h intervals. At each time, analysis of variance (ANOVA) and Bonferroni post hoc test were carried out to assess overall differences in O.D. readings obtained from different strains in relation to the control.

## Competing interests

The authors declare that they have no competing interests.

## Authors’ contributions

ADC and RE developed and performed the experiments by dynamic light scattering and drafted the manuscript. MA did the assays about MIC to HA, HA utilization and strains’ resistance to simulated gastric juice. MB and BP provided scientific orientation and revised the manuscript. All authors read and approved the final manuscript.
